# Effects of Environmental Enrichment on Pig Welfare—A Review

**DOI:** 10.3390/ani9060383

**Published:** 2019-06-22

**Authors:** Dorota Godyń, Jacek Nowicki, Piotr Herbut

**Affiliations:** 1 Department of Production Systems and Environment, National Research Institute of Animal Production, 32-083 Balice n. Kraków, Poland; 2 Department of Swine and Small Animal Breeding, Faculty of Animal Sciences, University of Agriculture in Krakow, 30-059 Kraków, Poland; j.nowicki@ur.krakow.pl; 3 Department of Rural Building, Faculty of Environmental Engineering and Land Surveying, University of Agriculture in Krakow, 30-059 Kraków, Poland; p.herbut@ur.krakow.pl

**Keywords:** enrichment, pigs, welfare

## Abstract

**Simple Summary:**

The legislation regarding pig housing systems states that environmental enrichment needs to be provided for group-housed pigs. Moreover, the materials used for improving animal housing are categorised as optimal, suboptimal, and of marginal interest. Straw has been considered as the optimal solution for pig housing, however there are some limitations in using it in a large amount. Therefore, other materials, objects, and toys have been used as enrichment in pig maintenance. Understanding how various enrichments influence animal welfare seems to be an important key to elaborating the best methods of improvement. This review presents new literature references regarding environmental enrichment for suckling piglets, weaning piglets, and fattening pigs.

**Abstract:**

Good husbandry conditions on farms is of key importance for assuring animal welfare. One of the most important legal documents regulating the rules of maintaining pigs is the Directive 2008/120/EC, which states that group-housed pigs should have access to litter or other materials that provide exploration and occupation. Released in 2016, the Commission Recommendation (EU) 2016/336 on the application of the Council Directive 2008/120/EC characterizes the various categories of materials that may be used to improve animal welfare. According to the document, straw is considered as an optimal material for pig housing, however, materials categorized as suboptimal (e.g., wood bark) and materials of marginal interest (e.g., plastic toys) are often used in practice and scientific research. As such, the aim of this paper is to review and systematize the current state of knowledge on the topic of the impact of environmental enrichment on pig welfare. This article raises mainly issues, such as the effectiveness of the use of various enrichment on the reduction of undesirable behavior—tail biting; aggression; and stereotypies at the pre-weaning, post-weaning, and fattening stage of pig production.

## 1. Introduction

In recent years, there has been a growing interest from consumers into food production. Taking into consideration products of animal origin, it may be stated that the public debate is focused not only on the good quality of such products. Currently, a great deal of attention has also been paid to maintaining good husbandry conditions on farms. Animal welfare qualities have been known for many years [1,2]. This term has been connected to many issues that are mainly related to the provision of a proper animal physical and mental state. Nowadays, there is a great deal of controversy over performing invasive procedures on farm animals, such as castration, teeth clipping, and tail-docking—especially when performed without anesthesia [3,4]. The pressure from consumers and animal welfare organizations regarding these and others aspects of animal rights is often reflected in legislation changes. At the same time, even the knowledge of professionals, official inspectors, and advisers in identifying the causes of tail biting and methods to reduce this behavior, as well as the understanding of legislation, is often insufficient [5].

One of the most important legal documents providing the minimum standards for pigs is the Directive 2008/120/EC [6]. This legislation states, among others, that pigs kept in groups should have access to litter or other materials that provide the possibility for exploration and occupation. Enrichment of the pigs’ environment is a way to reduce aggression and other pathological behavior [7]. One of the greatest problems in the intensive production of pigs is tail biting. Taylor et al. [8] described the different motivations for performing this behavior. The factors that may influence the expression of tail biting are as follows: improper diet, an absence or delay of food provision, gastrointestinal discomfort, poor health, genotype, too large density, and unfavorable microclimate conditions. An important factor that may lead to a higher frequency of this behavior is also the lack of a substrate or object to manipulate in the pigs’ surroundings [7,8].

In 2016, the Commission Recommendation (EU) 2016/336 on the application of the Council Directive 2008/120/EC laying down the minimum standards for the protection of pigs regarding the measures needed to reduce the need for tail-docking was released [9]. As the document states, this procedure should never be performed routinely. The Commission Recommendation provides useful information on estimating the risk of tail biting, and stipulates improving the conditions on the farm first, before invasive procedures become necessary. Taking into consideration the aspect of enriching the pigs’ environment, this document—more precisely than the directive [6]—describes and characterizes the various categories of materials that may be used to improve animal welfare. The four-point evaluation of the enrichment materials contained in the Commission Recommendation states that they should be edible, chewable, investigable, and manipulable. Moreover, as the document states that enrichment materials should be provided in such a way that they are of sustainable attraction for pigs (e.g., regularly replenished), they should be accessible for oral manipulation and should be provided in sufficient quantity. They should also be clean. Only if the materials meet all of the above-mentioned criteria, may they be considered as an optimal. Suboptimal materials possess most of the above-mentioned characteristics, and they should be combined with other materials in pig housing. The last group of enrichments are materials of marginal interest. They cannot fulfill all of the animal’s needs, therefore they should be used together with optimal and suboptimal materials.

In spite of the fact that straw is considered as the optimal material for pig housing improvement (according to the characteristics of different enrichment materials included in the recommendation), in slatted floor systems, it is not easy to introduce it, as it may lead to blocking the manure system [10]. However, enrichment devices commonly used in conventional farming, made of metal and plastic, may be assigned only as materials of marginal interest. Therefore, there is a great need to create and implement new materials and forms of environmental enrichment in pig housing. It also seems very important to analyze the results of the studies concerning the effect of various enrichment devices, and to check whether the materials used in the experiments meet the requirements contained in the Commission Recommendation. 

The aim of this paper is to review and systematize the current state of knowledge on the topic of the impact of environmental enrichment on pig welfare. This review covers the issues of using the various materials to improve the housing of different pig groups, piglets pre- and post-weaning, and fattening pigs. In this review, the latest scientific literature has been included, mainly concerning the effect of enrichment materials on the reduction of undesirable behavior, such as tail biting, aggression, and stereotypies. 

## 2. Welfare Problems in Pigs Resulting from Intensive Production Systems and the Methods of Welfare Assessment

### 2.1. The Concept of Animal Welfare and Assessment Methods

Animal welfare has been a scientific concept for many years [11]. It can be evaluated by scientific methods, and it may range from very poor to very good [12]. The term animal welfare covers, among others, the importance of the animal’s ability to keep control of mental and body stability in different environmental conditions [13]. Broom [1] has established the definition of welfare as a state of an individual with regards to its attempts to cope with its environment. Animal welfare is poor when behavioral, physiological, immunological, and other brain coordinated component coping strategies fail. Indicators of welfare based on an animal’s physiology and behavioral changes have been commonly used in recent years [12]. Among others, the presence of disease, injury, social interaction in the herd, housing conditions, and human handling are factors affecting animal welfare [12]. Improper social, hygienic, and microclimatic conditions may cause difficulties in maintaining the animal’s mental and physical stability. Although an individual may adapt to unfavorable conditions, it may still suffer—feeling pain or frustration. Thus, adaptation does not always mean good welfare [12]. Currently, positive welfare indicators have been increasing in popularity [12,14,15]. Three of these behavioral indicators, as a tool for welfare assessment, were proposed by McCormick [15], as follows: the indicator of contentment/pleasure, the indicator of luxury behaviours, and the indicator of behaviours that support the ability to cope with challenge. Moreover, positive emotions in pigs may be indicated by play, barks, and tail movement assessment, while negative emotions are indicated by ear movements, tail low, freezing, defecating, urinating, escape attempts, and high-pitched vocalisation [16]. 

The occurrence of undesirable behavior may be considered as an indicator of poor welfare [13,17]. In assessing animal welfare, the scoring of injuries and skin damage is a very important tool [18,19]. Skin lesions noticed on the front body part of pigs reflect the high frequency of fighting among individuals in the pen. In turn, skin damage found on the middle region of the pig body does not show a significant relationship with the occurrence of this behavior. Skin damage on the rear part (including the tail) indicates that the individual has spent a large proportion of time being bullied.

Besides behavioral indicators, physiological measures are very commonly used to assess animal welfare. Physiological indicators of animal welfare, which are often used in animal studies, are measurements of stress hormones [20]. Improper environmental conditions evoke an animal body response, such as alterations in the hypothalamo–pituitary–adrenocortical (HPA) axis, whose consequence is, among others, the release of glucocorticoids [21]. When discussing animal welfare, it may be stated that a more important issue is the situation of chronic rather than acute stress. Chronic stress is a situation when the animal is unable to cope with its environment. It is worth adding that according to Munsterhjelm et al. [22], a barren environment compared with an enriched one is associated with signs of chronic stress. The long-term elevation of cortisol levels leads to impairment deterioration of the physical and emotional state [23,24]. The measurement of cortisol in blood (serum) has been a popular tool for the assessment of animal welfare, but currently, the search is on for methods that are less invasive than blood sampling [25,26]. Animal handling and immobilization may prove a threat, and can themselves constitute a source of anxiety for an animal and, therefore, induce the secretion of stress hormones [25]. Glucocorticoids measured in saliva, faecal, or hair samples are also reliable methods for the activation of the hypothalamic-pituitary-adrenal (HPA) axis, and the sampling procedure of these materials is less invasive [27,28]. However, taking into consideration that stress responses consist of different systems within the organism, the cortisol level is just one of the physiological parameters that should be evaluated [29]. The review of Mkwanazi et al. [30] raises many issues linked with various enrichments in pig housing. Among others, the performance and blood metabolites in pigs kept in enriched and barren pens were discussed by the above-mentioned authors. There has been some evidence that environmental enrichment may have a positive effect on growth rate and lower creatine kinase [31,32].

A wide range of behavioural methods used together with, preferably, non-invasive biomarkers, is of key importance for the reliable assessment of animal welfare.

### 2.2. The Natural Behavior and Needs of Pigs

When discussing animal well-being concepts, it must also be stated that animals have strong motivational systems to meet their essential needs. These include basic needs for eating and drinking, but also for exploring the surroundings and expressing their natural behavior [12,33]. Rooting is one of the most important activities for pigs; it is performed to forage, sows root to build a nest, and rooting may also have some thermoregulatory functions [34,35]. It seems that the absence of materials for rooting, foraging, or manipulating causes the direction of the pig’s attention to be directed to other individuals in the pen [36,37]. According to some authors, the majority of pigs in Europe are reared in conditions of intensive production, in a barren environment with slatted floors [38,39]. This type of housing does not favor the expression of species-specific behavior. It has been shown that a lack of possibilities for being able to explore the surroundings in intensive production may lead to increased incidents of aggression, cannibalism, tail biting, and stereotypies [40,41,42]. 

Play may be considered as a luxury behavior, and thus as a positive indicator of welfare [15,16]. It is very important, especially for young pigs. Providing objects to play with in the piglets’ environment may improve their social skills. It has been suggested that animals reared in an environment that enables expression of play behavior are better prepared to cope with unfavorable situations at a later stage of life [43].

### 2.3. Risk Factors for Undesirable Behaviours under Intensive Conditions

Tail biting is a huge problem for both the economic and welfare aspects of pig production. Taylor et al. [8] identified three different forms of tail biting, namely: two-stage, sudden-forceful, and obsessive. Two-stage tail biting is a situation when pigs gently manipulate another individual’s tail, and at some point in this “routine”, the skin damage occurs. Fresh blood may attract other pigs to bite, and it consequently, may lead to increased aggression within the herd, and even cannibalism in some cases [44]. This form of tail biting may be prevented by providing some materials to develop a manipulatory interest. Another form of this behavior may be characterized as an acute and rapid attack performed without any previously gentle manipulation of the tail. It is often seen when pigs have to compete for access to water or food [45]. The obsessive tail biting is mainly represented by one or a few individuals. Tail biters seem to be focused on this body part, and continuously look for another one to grab or bite. The occurrence of this pathology may be linked with unsuitable feed and genetic lines, and obsessive tail biting may be seen as some form of stereotypies.

An increase in aggressive behavior is often observed during mixing, as this is generally considered a stressful procedure, when the individuals of different herds are placed together in one pen. The social hierarchy must be established, so it is often linked with fighting and aggression [46]. Only after the herd hierarchy is established, the fights among the individuals of the pen gradually decline [47,48]. 

Another form of behavior observed mainly in weaned piglets, especially in those weaned earlier, is the belly nosing phenomenon characterized by the rhythmical rubbing of a piglet’s snout on the other individual’s belly [49]. Widowski et al. [50] described in detail the various aspects related to the occurrence of this stereotypic behavior. One of the reasons for belly nosing or belly suckling is early separation from the sow. The performing of this behavior among piglets for a longer period of time often leads to lesions, and also may negatively impact the pigs’ performance [50]. These authors presented some evidence about the complex conjunction of belly nosing, drinking, feeding, and suckling in weaned piglets. Briefly, the strong motivation to suckle, together with an insufficiently developed ability to independently feed in early weaned piglets may be a reason for oral behavioral problems. As Breuer et al. [51] claimed, belly nosing may also have a genetic basis or may be a general behavioral reaction to stressful situations [52]. An environment with poor stimuli may also contribute to the development of this behavior [53,54]. 

There has been some evidence of how stereotypic behavior is induced in farm, laboratory, and wild animals kept in a barren environment [55,56,57]. Stereotypies are defined as a simple ritualized, non-functional, repetitive behaviors [58]. Situations in which the animal has a strong motivation to meet its needs, but cannot reach them, are frustrating. The results of this frustration may be reflected in substitute behavior or stereotypies [59]. Moreover, some stereotypies may be an attempt to reduce the physiological symptoms of stress. This behavior may lead to reduced anxiety and the ability to react to external harmful stimuli, as the animal’s attention is diverted from the source of the conflict [59]. Stereotypies is mainly linked with the functioning of the basal ganglia. Briefly, it may be assumed that the behaviors associated with the continuous repetition of activities have their source, among others, in the disturbed balance between the activation of the two main basal ganglia regulatory loops [60]. 

### 2.4. Environmental Enrichment as an Effective Prevention/Mitigation Strategy for Tail Biting

In animal models, it has also been shown that environmental enrichment for the prevention of stereotypies is linked with a higher level of neuronal metabolic activity and dendritic morphology (more dendritic spines) in the motor areas of the brain [61]. Taking into account the multi-factorial nature of tail-biting risk, the provision of good enrichment is an important starting point, but it may not be enough to prevent an outbreak of tail biting [62]. When assessing the level of well-being, it should be observed how pigs are involved in interacting with their environment. It is necessary to check whether pigs are able to express appropriate exploration/manipulation behaviors; whether the enrichment material is edible, chewable, and destructible; and whether pigs deal with playpen elements instead of enrichment objects/materials. According to the directive, it is also important to find out whether all pigs have permanent access to a sufficient amount of enrichment materials that is safe and clean (EUWelNet training tool). This evaluation may constitute the basis for the selection of the best environmental enrichment solutions.

## 3. Different Types of Enrichment

The first idea for enriching the environment was proposed for laboratory animals in the 1940s [63]. Since that time, many studies have been carried out regarding the positive effect of an improvement in the animal environment on its brain structure and biochemical changes [64]. Environmental enrichment should stimulate animals’ visual, somatosensory, and olfactory systems, and the key idea is that these objects should provide an aspect of novelty [65]. 

Taking into consideration the fact that pigs may lose attention towards the object within a few days [66], it is of key importance to sustain the animals’ interest by the frequent replacement or renewal of enrichments. Moreover, the pigs should have enough materials to play with at the same time. These explanatory notes were included in the aforementioned Commission Recommendation (EU) 2016/336 [9]. Based on different categories of enrichment, straw, but also other materials such as green fodder, miscanthus pressed or chopped, and root vegetables, when used as bedding, may be considered as optimal for animal welfare [9]. As van de Weerd et al. [67] claimed, fragrance, chewability, and deformability are features of objects that, at the beginning attract the pigs, however, the destructibility and edibility of the enrichment may attract them for a longer time. In the Commission Recommendation, peanut shells, fresh wood, corn cobs, natural ropes, compressed straw cylinders, shredded paper, pellets, and so on are considered as suboptimal. Moreover, straw provided in racks or in dispensers is treated as a suboptimal material as well [9]. The marginal materials include, among others, chains, rubber, soft plastic, pipes, hard woods, and balls. For the best results when bedding cannot be provided, the combination of different kinds of enrichment materials should be used [9]. 

It seems that in spite of the fact that many years have passed since the Council Directive 2008/120/EU was introduced, there are still some experiments carried out using objects and materials not admitted by this legal EU Act, showing some positive effect on pigs’ behavior. However, the earlier results obtained by Scott et al. [68] and Zwicker [69] indicate that straw bedding much more effectively ensures a high interest in this type of enrichment, compared with hanging toy(s), even when the number of hanging objects is increased. Moreover, straw provided in racks increases exploratory behaviour, especially when more enrichment of this type is provided. Hanging elements do not provide as much interest for pigs as straw, because they are not rootable, and most of them are not edible, so they do not fulfil the requirements from the recommendation. It is worth adding that the Recommendation, in comparison to the directive, contains many more valuable details.

The review of the literature included in the report of Ernst et al. [66] covers the majority of different solutions used for environmental enrichment in pigs’ conventional production. Some authors confirm the benefits of using different kinds of enrichments at the same time [66,70]. Before the Commission Recommendation 336/2016 was introduced into practice, the objects most often used were metal chains or commercially available plastic or rubber toys [71]. There is some evidence that this may still be the case. For example, the final report of an audit carried out in Germany from 12 February 2018 to 21 February 2018 in order to evaluate member state activities to prevent tail-biting and avoid routine tail-docking of pigs [72] showed that the German authorities can impose administrative fines for non-compliances with directives. But it was noted that some requirements of the provisions of the Council Directive 2008/120/EC are not directly sanctionable through this system, in the case of the characteristics and sufficient quantities of the enrichment material. Moreover, although central and land authorities have spent considerable sums on research and communicating its results, their strategies to reduce tail biting and avoid the routine tail-docking of pigs have not produced tangible results, and tail-docking is still routinely carried out in Germany. The central competent authority estimates the incidence of tail-docking in Germany is over 95%. In addition, the French Agency for Food, Environmental, and Occupational Health and Safety consider chains “acceptable” as an enrichment [73]. In addition to the optimal materials and objects listed in the Commission Recommendation, environmental enrichment may also be a larger space, fragrance, or even music [57,74,75]. Nutrition can be a special enrichment, especially the way it is provided to animals (e.g., hidden in the substrate) may have some key benefits for improving the pigs’ activity [16]. Nowadays, various forms of cognitive enrichment, linked mainly with a reward gained by an animal, may bring many advantages regarding pig welfare [76]. Reimert et al. [16] found that rewarding events both during training and testing in pigs are often linked with an increase in play behavior. The authors observed also that barking and tail wagging occurrence is more common in pigs during rewarding events. Thus, it may generally be considered that a reward gained by an animal is associated with eliciting positive emotions. However, it should always be remembered that the legislation requirements should be fulfilled.

## 4. Environmental Enrichment for Sucker and Weaned Piglets

The effects of environmental improvement have been studied in pre- and post-weaning piglets. It seems that providing the enrichment in early, neonatal housing conditions may lead to better social behavior at later stages of the pig’s life [75,77,78]. As has been shown, access to straw, wood shavings, wood bark, or even pieces of newspaper may have a positive effect on a piglets’ behavior at the pre-weaning stage [79,80,81]. 

The effect of straw provision at an early stage may be effective in the reduction of nosing and tail biting [77]. Access to straw at the pre-weaning period may also lead to a reduction in mounting and oral manipulation directed at other individuals, as well as positively affect piglets’ growth [80,82]. Moreover, Brajon et al. [82] observed more time spent lying in piglets that had access to straw in the pre-weaning period, which may be considered as a positive indicator of animal comfort.

Positive indicators of animal welfare may also be observed when materials other than straw are provided. As mentioned previously, belly nosing has a multifactor origin, and generally may be considered as an indicator of poor welfare in weaned piglets [82]. Previously, it was documented that straw provision may not be effective in the total elimination of this phenomenon [50,82,83]. However, some positive aspects of belly nosing reduction were found when black foam rubber matting was used [84]. Similarly, in other studies, the most effective influence on some of the behavioral and physiological indicators were materials classified according to the recommendation as suboptimal or of marginal interest [79,81]. Yang et al. [79] found that play behavior was performed more often by piglets of the two groups that had access to enrichment (hanging objects or wood bark) than individuals kept in farrowing pens without any improvement. In turn, in the study of Telkänranta et al. [81], all of the experimental animals were housed in pens with environmental enrichment. However, the individuals of the control group only had access to balls and wood shavings, and the piglets of the experimental group had access to sisal ropes, plastic balls, pieces of newspaper, and wood shavings. The sisal ropes and newspaper pieces in particular induced the pigs’ activity, as well as influenced a reduction of oral–nasal manipulation directed towards pen mates.

Further effects of environmental enrichment provided in the neonatal period were shown in some studies [75,80,81,82,83]. 

It is well known that chronic stress has an immunosuppressive effect [85]. Taking this aspect into consideration, it worth referring to the studies of Yang et al. [79] and Van Dixhoorn et al. [80]. The first mentioned authors found that piglets that had access to wood bark at the neonatal stage had a lower level of cortisol after weaning. In turn, the latter authors found that piglets kept in a pen with straw, moist peat, and wood shavings at the pre-weaning stage were characterized by a less severe onset of the reproductive and respiratory virus and *Actinobacilllus pleuropneumoniae* co-infection at a later stage of their lives.

In turn, Telkänranta et al. [81] reported that richer environmental enrichment provided in the neonatal period caused reduced severe tail damage (wounds with swelling and infection or partial/total loss of the tail). This effect was not seen in the Yang et al. [79] study. No significant impact on aggressive behavior was found; however, the aggression level was evaluated through the skin lesion pre-weaning, and two days after mixing. However, perhaps more days of observation were needed. Martin et al. [75] compared the behavior of piglets kept in standard neonatal housing conditions and in improved (larger and with straw provision) pens. These authors claimed that three days post-weaning, the aggression was similar, regardless of whether the enrichment had been provided or not, but after seven days, a significant drop in this behavior was observed in the enriched group.

Brajon et al. [82] found that the individuals that were stimulated during the neonatal period performed less nosing and chewing behavior towards pen mates than the piglets of the control group, six days after weaning. The situation changed after six days; belly nosing became significantly higher in this group and activity levels decreased. This was probably the effect of stress, linked with weaning and enrichment removal. 

As has been well documented in previous studies, the sudden limitation of enrichments may cause an increase in harmful social behavior [86,87]. This effect was noted in the Statham et al. [83] study. The authors carried out a longitudinal experiment, whose aim was to evaluate the impact of straw provided through the entire production cycle. The behavior of the animals assigned to four groups was observed. The first group was housed with straw through the entire production cycle (from farrowing to finishing pens); in the second group, straw was provided from weaning; and the third group was kept on straw only in the finishing pen. The fourth group had no straw provided; however, prior to weaning, the pens of the third and fourth groups had a small amount of wood shavings added. Taking into consideration the proportion of the diverse behavior categories performed by piglets at the pre-weaning stage, there was no significant difference between the treatments. Moreover, the authors claimed that access to straw throughout the animal’s life did not affect the level of aggression, belly nosing, or tail biting. This lack of differences between treatments may result from the pens’ construction, which enabled the adding of a greater amount of straw in the early stages than at the finishing stage. This could induce the aforementioned negative effect of enrichment removal in animals. Moreover, the lack of significant differences between treatments in this study could also be related to the insufficient frequency of straw provision (twice a week); novelty in the pigs’ surroundings may be ensured when straw is added more often [78]. In addition, it is worth mentioning that all of the piglets had access to enrichment (straw or wood shavings) at weaning, so possibly, the effect of both of these substrates was comparable.

Aromas in pigs’ surroundings may be a factor causing positive stimulation, which has been proven in some studies [84,88,89]. In the Bench and Gonyou [84] experiment, a soil-filled tray was very effective in the improvement of the weaned pigs’ manipulating behavior. The pigs were probably attracted by the smell of this enrichment. Nowicki et al. [88] found that among the synthetic and natural aromas, the weaned pigs preferred the most natural fragrances, for example moist soil, fresh grass, and dried mushrooms. Moreover, at the second stage of their experiment, the authors compared the behavior of pigs kept in three different pens. The first pen was without enrichment, the second had an aromatized object (firstly with moist soil, then with fresh grass), and in the third pen was placed a container without any aromas. The aromatized containers had the greatest impact on reducing the time spent on agonistic behavior during the first nine days of observation. After this time, there was no significant difference between the treatments. It was also stated that the possibility of aroma exchange after several days of use provides the required novelty, and makes the enrichment attractive again. In turn, Sartor et al. [89], in addition to different fragrances, used Vivaldi’s music and blue light emitting diode—LED lightening as enrichments in their study. The experiment was performed over 10 days on sucker piglets. Of the fragrances of chamomile, lavender, lemon, and thyme used in the heated creeps, the piglets preferred the latter aroma the most. Blue lightening was also an important factor that caused longer time spent in the creep by the piglets. The classical music stimuli, however, affected higher activity among the piglets. 

Other environmental enrichment than that mentioned previously was tested by Winfield et al. [90]. The authors evaluated different shapes of nutritional lick blocks on sucker and weaned piglets’ behavior. Taking into consideration the wedge-, brick-, and cube-shaped blocks, the authors found that the brick shape attracted the piglets the most. Multiple piglets would stand together to chew the block. This behavior was similar to co-operatively massaging a sow udder, thus the authors suggest that this shape of enrichment may be a factor evoking natural co-operative behavior. 

Weaned piglets experience multiple stressors while they are subjected to nutritional and social changes [91]. The literature references presented in this section show some evidence that the environmental stimuli may alleviate the negative effects of this treatment. Even the presence of suboptimal materials in the pen after weaning was shown to be effective at reducing harmful behaviour during the first period after mixing [88]. A key aspect also seems to be to provide the enrichment at the neonatal stage.

Table 1 shows some current literature references regarding enrichment for sucker and weaned piglets, and includes the main effects of each type of enrichment on pig behavior. It also shows the categories of environmental enrichment in reference to the Commission Recommendation.

## 5. Environmental Enrichment for Fattening Pigs

Taking into consideration the environmental enrichment of growing and finishing pigs, a larger space seems to be a key factor for providing animal comfort. In the study of Cornale et al. [39], the faecal corticosteroids level was found to be higher in the pigs kept in pens with a greater number of individuals, even when access to enrichment (cylindrical pieces of hard wood suspended on a chain) was provided. In turn, the experiment of Di Martino et al. [92] showed that at high stocking density conditions, the cases of tail lesions were high, even when the animals had access to straw racks. These results may suggest that a larger space is a very important factor that may reduce this pathology.

It is worth adding that the fattening heavy pigs used for specific productions require the provision of more space than the minimum legal requirements. These situations may be seen when pigs reach 180–190 kg of body weight, and when the fattening process is long [92]. The Council Directive 2008/120/EC states that pigs should benefit from an environment corresponding to their needs for exercise and investigatory behavior. The welfare of pigs appears to be compromised by severe restrictions of space. The 1 m^2^ space for pigs heavier than 150 kg seems to be insufficient. Moreover, the directive states that before carrying out tail docking, other measures should be taken in order to prevent tail-biting and other vices, taking into account environment and stocking densities. For this reason, inadequate environmental conditions or management systems must be changed. 

The genetic background is also a significant factor of tail biting occurrence [7]. This was confirmed in the study of Bulens et al. [93]. The authors compared the effect of two methods of enrichment—straw block and chain versus only chain provision—for finishing pigs of two genetic backgrounds. The boar type was the factor for determining the frequencies of tail biting, regardless of the enrichment provided.

Some positive aspects of the prevalence of mild damage to the tail and ear biting reduction were found in the Telkänranta et al. [70] study, by providing pieces of freshly cut birch trees. This enrichment was the most effective in the mentioned aspects, but the authors also tested different materials. The pens of the control group were provided with a straw dispenser, wood shavings, and metal chain; the four other groups had the same enrichments and some other materials such as a horizontally suspended piece of fresh birch wood, polythene pipes, and metal chains suspended vertically. The last group was housed in the pens equipped with all of the enrichment objects mentioned above. The other result of this study was the finding that freshly cut birch wood and polythene pipe, among other enrichment devices, significantly affected the frequency of object manipulation. 

Continued high interest in straw investigation was found in the study of Scott et al. [68], when this substrate was used as a bedding. However, the study of Nannoni et al. [94] confirmed that even when bedding is not provided, finishing pigs in all production cycles were interested in exploring the environmental enrichment inside the racks. These authors tested wooden logs and a vegetal edible block placed inside the metal racks compared to hanging chains. Taking into consideration the effect of enrichment materials on tail biting cases, the statistical analysis showed significant differences only between the moderate tail lesion noticed in the group with access to the wooden logs, compared to the group with the chain. Despite the authors’ suggestion that the vegetal edible block helped to reduce the exploratory behavior directed towards the pen floor, it seems it could not fully reduce the pigs’ attention directed towards other pigs. The group that had access to this enrichment was characterized with some moderate and severe tail lesions. This could be a result of competition for access to enrichment, as was also shown in the study of Bulens et al. [93]. In this experiment, mounting and fighting was higher at the beginning of the finishing phase in the pigs that had access to the straw dispenser. 

The effect of the enrichment on stress response in finishing pigs was tested in Casal et al.’s [28] and Nannoni et al.’s [94] research. In the Casal et al. [28] study, the pens of the experimental groups contained sawdust, natural hemp ropes, and rubber balls, whereas the control group was kept in a barren environment. The authors also tested herbal supplementation. After two months of treatments, the hair cortisol levels and salivary chromogranin A (CgA) were the highest in the control group. Consequently, the authors suggest that both enrichment and herbal compounds may lead to stress reduction in pigs. However, the enrichment devices used in Nannoni et al.’s [94] research had no impact on the hair cortisol levels.

The above-mentioned findings [28,93,94] were obtained through a long-term study. This way of performing an experiment seems to be very important for the reliable assessment of the impact of different categories of enrichment, especially when the markers of stress are used. However, the impact on the behavior of the environmental improvement was found during only six days of observation in the Machado et al. [95] study. One of the trials performed in this research was to evaluate the effect of the availability of hanging toys (made from a polyvinyl chloride—PCV pipes and plastic tubing). The pigs of the control group had no access to the enrichment, the individuals of the first group had the enrichment for the experimental period, the pigs of the second group had the objects in their pens but provided on alternate days, and the pigs of the last group were provided with the objects in the morning and then had them removed in the afternoon. The results showed that the individuals in the pen without enrichment and those that had access to the objects for the entire time spent more time eating and drinking. However, as suggested by the authors, the individuals of the pen without objects might have wasted the food during play. The higher percentage of sexual behavior (mounting) was found among the pigs with access to the enrichment provided on alternate days. It could be the same effect as seen in previously presented studies [93,94]; when access to enrichment is limited, competition rises, and consequently, a negative form of behaviour may occur. In the Machado et al. [95] study, resting behavior was performed the most frequently in the group with no object, compared with individuals that had access to enrichment constantly or on alternate days. There were no differences, however, between the treatments regarding the time spent on interacting with other pigs, nuzzling, or exploring the environment. Moreover, the objects had no impact on agnostic behavior, which generally was quite low during the experimental period. 

One of the main findings of this study was the increase of animal activity, even when only the material classified by the EU Recommendation as being of marginal interest is provided. The animals of the control group which had no access to a “toy” remained for the majority of time inactive. Based on this and other results presented in this section, regardless of fact that harmful behavior still occurred in the pigs kept with access to enrichment, it may be stated that the increase of animal activity and interest in an enrichment object is very valuable from an animal welfare perspective. Moreover, Scott et al. [68] pointed out the greater benefits of using straw as an environmental improvement to hanging toys; however, in the Telkänranta et al. [70] study, it was proven that fresh wood attracted pigs the most, even when, at the same time, they had access to straw. These kinds of results are promising for pigs housing, where the technological system of maintenance makes straw provision limited or simply impossible. On the other hand, it should be stated here that in practice within EU countries, enrichment materials classified as being of marginal interest cannot be used alone (without provision of optimal enrichment).

Similarly, as in Table 1, Table 2 shows some literature references regarding enrichment, but for fatteners, including the main effects of each type of enrichment on the behavior of pigs. It also shows the categories of environmental enrichments in reference to the Commission Recommendation.

## 6. Conclusions

Based on the various research results presented in the review, it may be stated that the enrichments provided for young and fattening pigs have many benefits with regard to animal welfare. Generally, it may be concluded that access to even a small amount of substrate and objects increases targeted activity. As it has currently been proven, enrichment given at the neonatal stage (farrowing pen) improves manipulating skills, and may also lead to better social behavior performed at later stages of the animal’s life. The main effects of enrichment may be seen through a reduction of attention paid to other individuals in the pen. This also includes the pigs’ interest in performing tail chewing, biting, or belly nosing. However, the results of a reduction in these harmful behaviors through the provision of enrichments is inconclusive. Despite the fact that studies have confirmed the advantages of using different kinds of enrichments at the same time, some results have also shown that only the use of materials that are considered to be of marginal interest (according to Commission Recommendation (EU) 2016/336) brought about the desired effect on the pigs’ behavior. It can be misleading for farmers, because it is strongly highlighted in the recommendation that “materials of marginal interest—materials providing a distraction for pigs, which should not be considered as fulfilling their essential needs, and therefore optimal or suboptimal materials should also be provided”. Moreover, some authors found a positive impact of materials, such as newspaper or PCV pipes, with protruding tubes. As such, it remains an open question whether these enrichments could be considered as completely safe for animal health; similarly for hard wood, whose chewing may also result in injury.

It is not possible and it makes no sense to change the European legislation regulating the types of environmental enrichment materials and their features. The current EU regulations are based on research results and represent the results of the European Union scientific projects. It should always be taken into account that tail biting and ear biting have a multifactorial origin, which is often difficult to determine. Tail-docking should be an absolute last resort (in some of the EU countries it is forbidden), so looking for alternative possible ways in terms of scientific and practical activities to stop and prevent tail biting should be essential (but always in agreement with the legal regulations). Making changes in the pigs’ environment, such as reducing stocking density and introducing optimal environmental enrichment solutions based on the scientific evaluation of their efficiency, should lead to the situation in which the procedure of tail-docking would be performed as little as possible, or not at all.

## Figures and Tables

**Table 1:** Environmental enrichment materials and objects according to the categories included in the Commission Recommendation (EU) 2016/336, and their main effects on sucker and weaned piglets’ behavior.

Animal	Enrichment	Duration of Behaviour Observation	Main Effect	References
sucker/weaned piglets	black foam rubber matting ***	8 h daily on 3, 10, 19, 26, 33 d of pig’s life	reduction in belly nosing	Bench and Gonyou [84]
sucker/weaned piglets	farrowing pen—straw bedding *	6 h daily on 6, 12, 20, 21, 22, 27 d of pig’s life	increased level of exploration, animal comfort improved	Brajon et al. [82]
sucker/weaned piglets	farrowing pen—wood shavings **, sisal rope **, plastic ball attached to chain ***, newspaper **	4 h daily one day per 2, 3, 9 wks. of pig’s life	at pre-weaning stage—decrease in pen mate manipulation	Telkänranta et al. [81]
	weaners pen—wood shavings **, sisal rope **, plastic chewing toy ***		at post-weaning stage—decrease in severe tail damage	
sucker/weaned piglets	farrowing pen—wood bark in a plastic box **	6 h daily on 4, 5, 11, 12, 19, 23 d of pig’s life	baseline of cortisol level on d 1 post-weaning	Yang et al. [79]
weaned piglets	aromatized plastic container **	24 h per 9 d after weaning	decrease of duration of agonistic behavior	Nowicki et al. [88]

* optimal, ** suboptimal, *** marginal materials according to the categories included in the Commission Recommendation (EU) 2016/336 on the application of the Council Directive 2008/120/EC, laying down the minimum standards for the protection of pigs with regards to measures to reduce the need for tail-docking [9].

**Table 2:** Environmental enrichment materials and objects according to the categories included in the Commission Recommendation (EU) 2016/336, and their main effects on fattening pig behavior.

Animal	Enrichment	Duration of Behavior Observation	Main Effect	References
fattening pigs	straw bedding * compared to slatted floor pens with different number of hanging toys ***	6 h daily in the week of entry, the week before the group size reduction and a week before slaughter	decrease of pen mate manipulation and increase of investigation of straw bedding	Scott et al. [68]
fattening pigs	straw rack**, metal chain***, wood shavings**, and additional pieces of fresh birch wood**, polythene pipes***, or two crosses of metal chain***; combination pens had all of the enrichment materials mentioned above	after 2.5 months of exposure to the objects, one day of video observation was performed	increased object exploration best seen in pens with fresh wood, as well as reduce of tail and ear biting	Telkänranta et al. [70]
fattening pigs	piece of hard wood attached to chain **	3 h daily, nine times over 16 weeks	decrease of aggression and tail biting	Cornale et al. [28]
fattening pigs	chain *** and compressed straw block in dispenser *	2.5 h daily, once a week through the entire finishing stage	decrease of pen mate manipulation	Bulens et al. [93]
fattening pigs	PCV pipes with four pieces of plastic tubing attached to it ***	8 h daily, for six consecutive days	increase of activity	Machado et al. [95]

* optimal, ** suboptimal, *** marginal materials according to the categories included in the Commission Recommendation (EU) 2016/336 on the application of the Council Directive 2008/120/EC, laying down the minimum standards for the protection of pigs with regards to measures to reduce the need for tail-docking [9].
